# Three decades of heart transplantation in the Netherlands

**DOI:** 10.1007/s12471-017-1015-y

**Published:** 2017-07-04

**Authors:** Y. M. Pinto

**Affiliations:** Department of Cardiology, Academic Medical Center, Amsterdam, The Netherlands

In this issue of the journal, Sammani et al. describe 30 years of heart transplantation in Utrecht [1]. It is remarkable to witness how this once exotic technology is now incorporated in our practice and an impressive account of how a dedicated team has been able to secure this technology in the care of end-stage heart failure patients.

Any form of organ transplantation still evokes that feeling of a high, innovative technology. Heart transplants are no exception. It is a therapy that is used only very rarely, and hence it is by definition very special. However, although hard to imagine it has been around so long it can be regarded as part of mainstream medicine. As we are all well aware, the first human heart transplant was done by Dr Christiaan Barnard already in December 1967, when he transplanted the heart of a 23-year old brain-dead woman into a 57-year-old recipient. Three years later more than 150 heart transplants had been performed worldwide. However, in these early days the ability to match donor and recipient was limited and the immune reaction could only be suppressed by corticosteroids. Hence, most – if not all – transplantations in those days failed quickly so that widespread disappointment made most abandon this idea altogether. As with many novel technologies, an additional innovation was needed to make organ transplantation feasible. This was the development of stronger and more selective immune suppression. Cyclosporine was discovered and developed by Jean Borel and colleagues in the 1970s [2]. Since cyclosporine inhibited T‑lymphocytes specifically and reversibly, immune suppression improved enormously. In the 1980s, cyclosporine impressively improved the success rate of heart transplantation and from there on the technology gained clinical acceptance.

It is, therefore, not surprising that in that decade the first heart transplant in the Netherlands took place at the Dijkzicht Hospital in Rotterdam in 1984, so that we have now seen more than 30 years of heart transplantation in the Netherlands.

As with many big strides in science, behind the fame and recognition of the first heart transplant is the race between competing researchers with often strange twists of fate that decide its outcome.

In fact, the same Christiaan Barnard had spent weeks in the laboratory of the American surgeon Richard Lower to watch how Lower had developed his technique to transplant hearts between dogs. Lower and his research partner, Dr Norman Shumway, had been close to performing the first heart transplantation on a number of occasions, but had to cancel due to donor-recipient incompatibilities. Back in South-Africa, Barnard spent months trying to establish Lower’s technique in his own lab, but the dogs he transplanted would die within days. Nevertheless he went ahead and did the human transplant anyway, while Lower and Shumway had to wrestle with more stringent transplant legislation in the US. And, as we know, although the recipient unfortunately survived the transplant only briefly, Barnard was designated the pioneer of human heart transplantation.

Adding to the irony was that Lower later had to face a million dollar lawsuit for allegedly taking out the heart of a donor too soon (a lawsuit he eventually won). This also illustrates the other side of the coin , where ethical issues are clearly a major part of the debate surrounding the technology. For instance, in the early 1990s, there was no legislation on brain death in Japan and heart transplantation was still strictly prohibited there [3].

In the Netherlands, the two main centres for heart transplantation are Utrecht (with around 5 transplants done yearly in Groningen) and Rotterdam. Together around 50 transplantations are performed yearly and it is clear that this number does not suffice to serve the population of patients with end-stage heart failure.

With the improvements made in assist devices, they are quickly taking an important place not only to gain time as patients await their transplantation, but increasingly as destination therapy.

As a result it is justified to wonder if we will still be transplanting hearts in 2048. When the external drive-lines of assist devices are no longer needed, and assist devices go through the next rounds of improvements, major problems such as infection, thrombosis and bleeding may be better managed. Then these artificial hearts may even replace heart transplantation all together and heart transplantation will again be overtaken by the next wave of innovation.
